# Current status of intra-cranial stereotactic radiotherapy and stereotactic radiosurgery in Australia and New Zealand: key considerations from a workshop and surveys

**DOI:** 10.1007/s13246-022-01108-4

**Published:** 2022-02-03

**Authors:** Lauren Pudsey, Annette Haworth, Paul White, Zoe Moutrie, Benjamin Jonker, Matthew Foote, Joel Poder

**Affiliations:** 1 Centre for Medical Radiation Physics, University of Wollongong, Wollongong, NSW Australia; 2 Institute of Medical Physics, School of Physics, University of Sydney, Sydney, Australia; 3 Nelune Comprehensive Cancer Centre, Prince of Wales Hospital, Sydney, NSW Australia; 4 Department of Radiation Oncology, Mater Hospital, GenesisCare, Crows Nest, Sydney, NSW Australia; 5 RPA Institute of Academic Surgery, University of Sydney, Sydney, Australia; 6 University of Queensland, Princess Alexandra Hospital, ICON Cancer Care Queensland, Southport, Australia; 7 St George Hospital Cancer Care Centre, Kogarah, NSW Australia

**Keywords:** Stereotactic radiosurgery, Stereotactic radiotherapy, Australia, New Zealand, Survey

## Abstract

Recently, there has been increased interest worldwide in the use of conventional linear accelerator (linac)-based systems for delivery of stereotactic radiosurgery/radiotherapy (SRS/SRT) contrasting with historical delivery in specialised clinics with dedicated equipment. In order to gain an understanding and define the current status of SRS/SRT delivery in Australia and New Zealand (ANZ) we conducted surveys and provided a single-day workshop. Prior to the workshop ANZ medical physicists were invited to complete two surveys: a departmental survey regarding SRS/SRT practises and equipment; and an individual survey regarding opinions on current and future SRS/SRT practices. At the workshop conclusion, attendees completed a second opinion-based survey. Workshop discussion and survey data were utilised to identify areas of consensus, and areas where a community consensus was unclear. The workshop was held on the 8th Sept 2020 virtually due to pandemic-related travel restrictions and was attended by 238 radiation oncology medical physicists from 39 departments. The departmental survey received 32 responses; a further 89 and 142 responses were received to the pre-workshop and post-workshop surveys respectively. Workshop discussion indicated a consensus that for a department to offer an SRS/SRT service, a minimum case load should be considered depending on availability of training, peer-review, resources and equipment. It was suggested this service may be limited to brain metastases only, with less common indications reserved for departments with comprehensive SRS/SRT programs. Whilst most centres showed consensus with treatment delivery techniques and image guidance, opinions varied on the minimum target diameter and treatment margin that should be applied.

## Introduction

For many years radiation therapy for small tumours and surgical cavities within the brain was confined to a limited number of treatment centres with specialized equipment [1–3]. Treatment was managed by expert multi-disciplinary teams including neurosurgeons, radiation oncologists and medical physics specialists trained for high precision therapy [1]. For linear accelerator (linac)-based treatments, patients were typically fitted with an invasive head frame to provide accurate target positioning and patient immobilisation [4–6].

With the growth of extra-cranial stereotactic ablative radiotherapy (SABR) in Australia and worldwide [7–13], there has been an increased interest in using a conventional linac and associated radiotherapy equipment for linac-based stereotactic radiosurgery (SRS) or stereotactic radiotherapy (SRT). Many standard treatment planning systems (TPS) now have the ability to plan highly conformal treatments of single as well as multiple lesions either individually or simultaneously in single-isocentre multiple-target (SIMT) SRS [14]. In addition to recognising that conventional technology could potentially be adapted for use with cranial SRS or SRT, there has been a change in referral patterns favouring a stereotactic approach for multiple brain lesions over standard whole-brain radiotherapy treatments in an effort to improve quality of life in patients with an extended life expectancy [15, 16].

With the demand for SRS and SRT increasing and the recognition that standard radiotherapy equipment could be adapted for precision therapy of small cranial targets came the question—when should patients be referred to a specialist centre because the standard equipment is unsuitable? In September 2020, the NSW/ACT Branch of the Australasian College of Physical Scientists and Engineers in Medicine in collaboration with the University of Sydney hosted a one-day workshop to determine medical physics community opinion on best practice for SRS and SRT. Two pre-workshop surveys collected data to understand the diversity of practice and opinions across Australia and New Zealand (ANZ). The first survey, intended to be completed by one representative from each treatment centre, collected data related to equipment and treatment protocols. The second survey was intended to define the diversity of opinion on SRS/SRT treatment delivery methods. During the workshop, clinical and medical physics experts from Australia and New Zealand provided evidence-based opinions on issues related to safe delivery of SRS/SRT treatments. An experienced SRS neurosurgeon (BJ) and radiation oncologist (MF) presented an overview of the clinical rationale for changes in clinical referral patterns and their opinions on the demand for future SRS/SRT services. At the conclusion of the workshop, the participants were asked to again complete the opinion-based survey so that the effect of community discussion and presentation of evidence from SRS/SRT experts could be assessed. In this manuscript we provide a summary of the evidence presented at the workshop, the results of the pre-workshop survey of equipment and treatment policies, and the outcome of the comparison of the pre- and post-workshop surveys.

## Methods

Prior to the workshop, two surveys were conducted using the online survey tool SurveyMonkey (SurveyMonkey inc., San Mateo, California, USA). The first survey was to be completed by one representative per department, and included 20 questions, specifically related to SRS/SRT, on topics such as: techniques and equipment used in the treatment planning, quality assurance (QA), and delivery of SRS/SRT. The second survey included six questions to be completed by individual medical physicists from these centres regarding opinions on current and future SRS/SRT practices.

The workshop was held on the 8th Sept 2020 via Zoom (Zoom Video Communications Inc., 2016) due to pandemic-related restrictions on travel and was attended by 238 radiation oncology medical physicists from 39 departments across Australia and New Zealand. Presentations throughout the workshop fell into three categories: clinical overview of the past, current and potential future SRS/SRT referral patterns from a neurosurgeon and radiation oncologist’s perspective; a comparison of approaches to SRS/SRT delivery from medical physicists; and research including clinical trial considerations.

Towards the workshop conclusion, an interactive presentation tool Mentimeter (Mentimeter AB, Stockholm, Sweden) was used to again collect responses regarding opinion on how SRS/SRT should be delivered. An expert panel was asked to discuss the results and take questions from the audience.

## Results

A total of 32 responses to the departmental survey were received; with 26 from Australian centres, three from New Zealand and a further three departments who remained anonymous. These responses represent 48% of departments currently in ANZ (combining satellite departments from the same city) [17]. To preserve the anonymity of the limited number of responding departments from New Zealand, data was not divided by country. A further 89 responses to the pre-workshop individual survey were received, of which four surveys did not include answers to all questions. The post-workshop survey was answered by 142 attendees of the workshop with eight surveys being incomplete. Responses to all surveys are summarised below.

### A. Equipment and departmental policy

#### The changing landscape of SRS in Australia and New Zealand

**Fig. 1:**
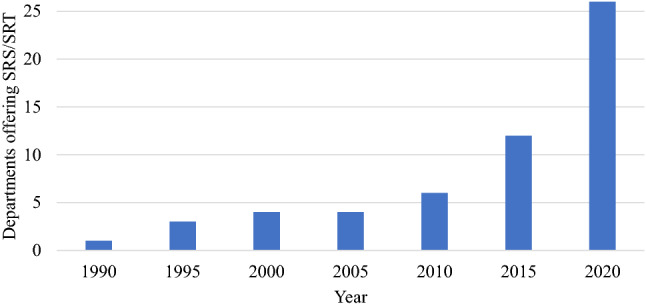
Cumulative total of departments offering SRS or SRT services over time based upon departmental survey results (32 respondents)

**Fig. 2:**
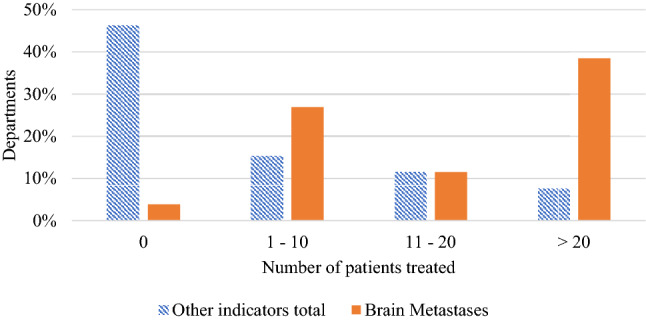
Number of brain metastases patients treated in 2019 by the 26 departments who offer an SRS/SRT service compared to total other indicators treated including AVM arteriovenous malformation, Acoustic Neuroma, Trigeminal Neuralgia, GBM glioblastoma

It was found that 26 of 32 respondents (81%) currently offer an SRS or SRT service. All departments which do not currently offer SRS/SRT plan to in the near future. Figure 1 shows the recent rapid growth in the number of departments offering SRS/SRT over time. The proportion of departments providing an SRS/SRT service who identified as regional/rural did not differ to the proportion of other departments providing these services. As illustrated in Fig. 2, brain metastases are by far the most common indicator for SRS/SRT, however, only 38% of departments who offer an SRS/SRT service treated more than 20 patients for brain metastases in 2019 using SRS/SRT.

#### Current delivery practices

**Fig. 3:**
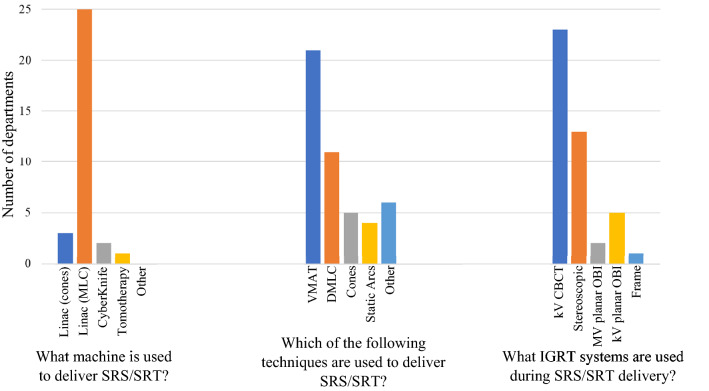
For the following survey results, more than one response was allowed: SRS/SRT treatment delivery device (31 responses), techniques used to deliver SRS/SRT (47 responses) and IGRT techniques used in SRS/SRT delivery (45 responses)

The majority of departments reported using linac based SRS with multi-leaf collimators (MLCs) (Fig. 3a) and of these departments 60% use a standard MLC width of 5 mm. The most commonly used techniques to deliver SRS/SRT include volume modulated arc therapy (VMAT) and dynamic MLC (DMLC) (Fig. 3b). A variety of image guided radiotherapy (IGRT) techniques are used (Fig. 3c). Only one department reported using a frame-based technique. This department also reported using kV cone beam CT (CBCT) and kV planar on-board imaging (OBI). All departments use a six-degrees-of-freedom (6DOF) couch to correct for patient positioning relative to the treatment plan.

**Fig. 4:**
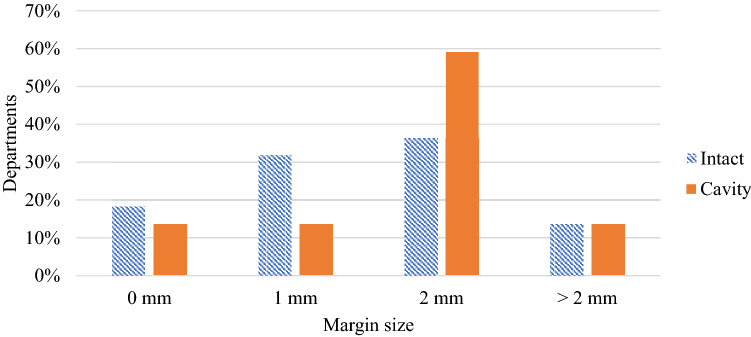
Survey results regarding PTV margins used for SRS/SRT treatments (22 respondents)

There was a wide range of responses to the question regarding the smallest planning target volume (PTV) diameter treated (0–40 mm), with most departments setting a limit at between 6 and 10 mm. Similarly, there was a range of PTV margins reported, with 2 mm the most commonly applied margin, with 36% and 59% of centres using a margin of 2 mm for intact targets and cavities respectively (Fig. 4).

#### Single-isocentre multiple-target SRS

**Fig. 5:**
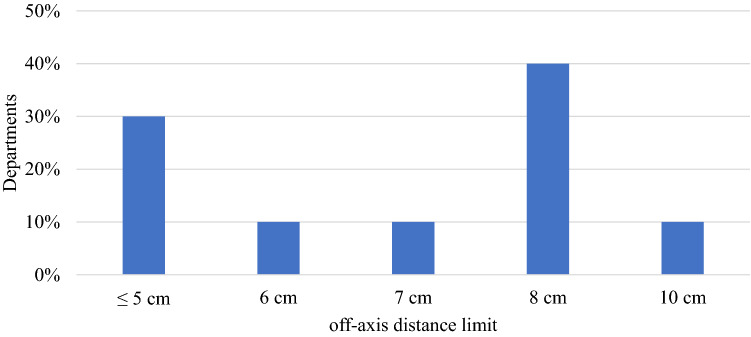
Off-axis distance limits used by those departments who limit the off-axis target distance away from isocentre in SIMT SRS (10 respondents)

Of departments delivering SRS/SRT, 72% offer SIMT SRS and over half of these (58%) do not limit off-axis target distance. For those departments with an off-axis distance limit, a range of limits were reported with 8 cm being the most commonly used (Fig. 5). For the majority of these departments (80%), this limit was not related to MLC width, i.e. confined to central MLCs in linacs with central MLC banks having a smaller width compared with outer MLC banks.

#### QA methodologies

**Fig. 6:**
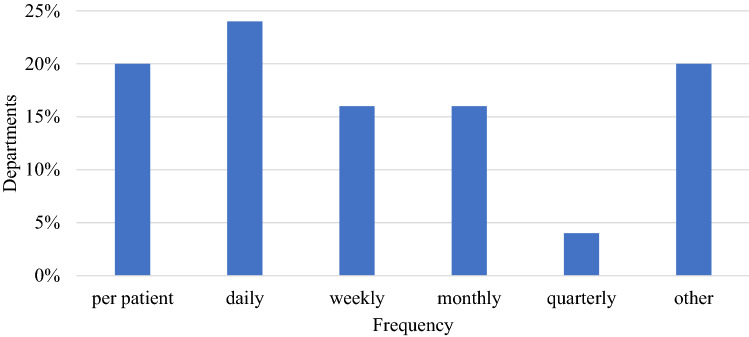
Frequency of performing a Winston-Lutz style test (25 respondents)

All but one department reported using a Winston-Lutz style test. The frequency of testing varied significantly as shown in Fig. 6. Respondents also reported combinations of per patient and weekly/monthly tests, as well as partial/mini tests followed by full tests if tolerances are exceeded. All but three departments reported some form of measurement-based patient specific QA. Of the departments performing measurement-based QA, 76% sample all high dose regions in SIMT SRS plans.

### B. Pre- and post-workshop individual surveys

**Fig. 7:**
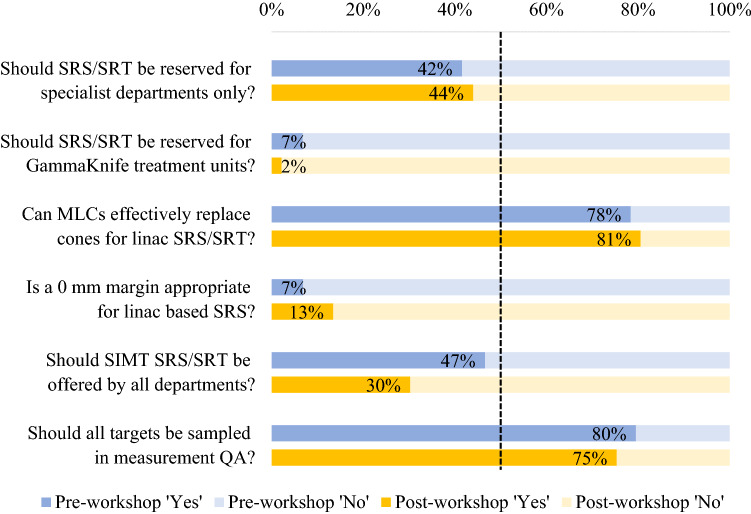
Individual pre- and post-workshop survey results

For most questions included in the individual response survey, there was little difference between pre- and post-workshop results (Fig. 7). Opinions were divided on whether SRS/SRT should be offered by all departments, however there was a strong consensus that SRS/SRT should not be reserved for Gamma Knife® units only.

There was a general consensus that MLCs can effectively replace cones for linac based SRS/SRT. Similarly, the majority of respondents agreed that a 0 mm margin was inappropriate, though the post-workshop survey indicated a small increase in support of a 0 mm margin (7% vs 14% accept a 0 mm margin may be used based on the pre- and post-workshop surveys).

The pre-workshop survey identified divided opinions on whether all departments should offer SIMT SRS. The post-workshop survey however, indicated that only 30% of respondents agreed that SIMT SRS should be universally available. The pre- and post-workshop surveys showed little change in opinion of whether all high-dose regions should be subject to quality assurance review (compared with a subset) with the results closely reflecting the current practice of 76% of departments sampling all high dose regions.

## Discussion

The motivation of this workshop was to obtain an understanding of the current status of SRS/SRT in ANZ. The workshop provided an opportunity for discussion regarding the changing referral patterns favouring the use of SRS/SRT and the growing use of conventional linac-based systems being used for treatment of multiple intracranial targets with one or more isocentres. From the workshop, and associated surveys, areas of consensus of opinion were identified, as well as areas where a community consensus was less clear. For those areas of practice where there is a lack of community consensus, expert groups could be charged with making recommendations and/or practice guidelines.

### The changing landscape of SRS in Australia and New Zealand

Recently there has been widespread growth in the use of SABR [7–9, 11–13] for extracranial disease using conventional linacs. For example, SABR is now considered the standard of care for medically-inoperable early stage non-small cell lung cancer [7–9, 18] and has shown increased use in other sites such as the spine and liver [12, 19]. With this growth in SABR, a similar trend of recent and rapid growth has been observed for the treatment of intracranial disease using SRS/SRT [20–25]. As shown in Fig. 1, the survey data presented here confirms this trend in ANZ with increasingly rapid uptake of SRS/SRT services between the years 1990–2020. Similarly, the findings of an Australian population-based study undertaken by Ong et al. [21] using data from the Victorian Cancer Registry and the Victorian Radiotherapy Minimum Data Set showed that of patients receiving radiotherapy for brain metastases, the proportion receiving SRS increased from 27% in 2012 to 35% in 2017. With growing confidence and experience in SRS/SRT it can be expected that referral patterns will continue to increasingly favour this method of treatment.

Of the respondents to this survey, already a majority (81%) of departments offer SRS/SRT with all others intending to implement it soon. However, it must be questioned at what point should SRS/SRT treatments be reserved for specialist departments or should all departments offer such a service. From Fig. 2, it can be seen that most responding departments (62%) provided ten or less SRS/SRT treatments for indicators other than brain metastases in 2019 including arteriovenous malformation (AVM), acoustic neuroma, trigeminal neuralgia and glioblastoma (GBM). In comparison, brain metastases are by far the most treated indicator. However, only 38% of departments who offer SRS/SRT treated > 20 patients for brain metastases in 2019. From the opinion-based pre- and post-workshop surveys, there is no clear consensus on whether SRS/SRT should be reserved for specialist departments. Based on the workshop discussion, there was general agreement that most departments could safely offer an SRS/SRT service, but only for more common indicators, namely brain metastases, with referral pathways for complex and less common treatment sites. These referral pathways may be dictated by the availability of specialised equipment for indications such as trigeminal neuralgia that are unsuitable for treatment with conventional MLCs or specialised imaging (for indications such as AVM), and access to support services.

Additionally, it was suggested that a minimum case-load of SRS/SRT treatments per year should be recommended, ensuring that the specialised skills of the multi-disciplinary team are maintained. The decision to offer an SRS/SRT service, and the corresponding minimum caseload would depend on availability of training, peer-review, resources and equipment, recognising that treating and planning with a standard linac and TPS may require additional commissioning and QA tests along with planning modules to accommodate single-isocentre multi-target planning.

Whilst the use of linac-based treatments (especially with MLCs and the SIMT technique) allows for the majority of radiation oncology departments to offer an SRS/SRT service and treat patients in an efficient manner, these techniques have a disadvantage that the low- and intermediate-dose wash to healthy brain tissues is larger compared to cone-based linac and Gamma Knife® treatments [26, 27]. Studies have shown that patients with some indications such as non-small cell lung cancer being treated with SRS/SRT now have a median overall survival > 2 years [28, 29], careful attention must therefore be paid to normal brain dose. Whilst it is possible to optimise this normal brain dose using advanced inverse planning techniques, the quality of the plan may be heavily dependent on the ability of the planner. This underscores yet again the need for a minimum case load for those offering an SRS/SRT service, and emphasizes the advantage of using cone-based linac, or dedicated systems such as Gamma Knife®, CyberKnife® or the recent ZAP-X® for benign indications, when minimising healthy brain dose is of upmost importance.

### Current delivery practices

Due to technological advancements, delivery of SRS/SRT using standard linac-based systems has become increasingly more common in recent years [2, 14, 26]. This survey found that linac-based SRS was by far the most used equipment for SRS/SRT delivery comprising (81%) of responses. This result was reflected in the opinion-based survey with a strong consensus that SRS/SRT does not need to be reserved for Gamma Knife® treatment units. This opinion remained unchanged after the workshop which included discussion of the comparable patient outcomes of linac-based treatments versus Gamma Knife® in several recent studies [26, 30, 31]. Also discussed were the lower associated expenses and convenient access of linac-based SRS over Gamma Knife® [2].

Whilst historically stereotactic cones were used in place of MLCs for linac based SRS/SRT treatments, our survey indicated that 81% of departments offered linac-based treatments using MLCs, with cones used in only 10% of departments. Of the respondents using MLCs, most departments (60%) are using standard 5 mm MLC width which again demonstrates a community perception that standard linac equipment can be used to safely deliver SRS/SRT treatment. The acceptance of standard MLCs is reflected in the recent consensus statement by Hartgerink et al*.* [6] with the recommendation of using MLC widths of ≤ 5 mm for linac-based SRS. The opinion-based pre- and post-workshop survey results showed a consensus (78% and 81% respectively) that MLCs can effectively replace cones. The recent practice guidelines for SRS/SABR presented by the American Association of Physicists in Medicine (AAPM) and the Radiosurgery Society (RSS) [32] includes recommendations for the use of both cones and MLCs, again indicating that MLCs are now widely accepted. Opposition to this statement throughout the workshop discussion mostly arose from cones holding an advantage in conformity for very small targets [33]. It can be suggested that MLCs may be able to replace cones for brain metastases, however for treatments requiring extremely high precision, such as trigeminal neuralgia, cones may be required.

The minimisation of healthy brain dose also brings attention to target margins used for linac SRS. The pre- and post-workshop surveys reflect an opinion that a 0 mm margin is not acceptable. However, a strong consensus in what margins should be used is currently lacking. The departmental results found 2 mm to be most commonly used for both intact targets and cavities though, as highlighted in the workshop discussion, recent studies have shown that increasing margins above 1 mm for intact lesions shows no change in local control but does increase risk of radionecrosis [34, 35]. A recent review of published literature in Australia similarly reported mixed results of GTV-PTV expansions though no specific recommendation was made [20]. This remains an area where a consensus is lacking and an opportunity for recommendations to be developed through clinical trials, with careful consideration of the accuracy and precision of target localisation and immobilisation equipment. These recommendations may also include different margins for SIMT SRS opposed to traditional SRS/SRT.

### Single-isocentre multiple-target SRS

MLCs offer an additional advantage over cones in that multiple targets may be treated with a single isocentre in a single treatment session using a SIMT technique. This allows for faster treatment delivery times as compared to cones, and therefore less time on the linac couch for the patient. The SIMT technique however does necessitate the need to correct for rotational patient setup errors, due to the targets often being situated many cm’s from isocentre [36]. A specialised six degree-of-freedom couch is therefore an essential component of a conventional linac being used for SIMT SRS treatments [6]. Encouragingly, all of the departments responding to the survey used a 6DOF couch for their SRS treatments.

### QA methodologies

With the increased treatment efficiency of SIMT SRS comes the need for lengthy QA procedures. This workshop raised the question; should all targets in the plan be sampled in measurement-based QA? Pre- and post-survey results showed that 80% and 75% (respectively) of individuals agree. However, SIMT is a relatively new technique and throughout the workshop discussion it was suggested that as confidence in equipment and delivery is built through a track record of excellent patient specific QA results, departments may decide that QA could be limited to a subset of targets. The main driving force for this would be the reduction in QA time, especially with the growth of SIMT and SRS/SRT in general increasing the workload of the medical physicist.

### Limitations and improvements

The departmental survey results presented here are representative of 48% of departments in ANZ and therefore some bias may be present. Although the response rate was low this was not unexpected as we are aware that many centres have well established referral patterns for SRS/SRT. Additionally, the survey did not request data related to training, experience and multi-disciplinary involvement. We suggest such information be included in future surveys to further support recommendations for complex treatments involving potentially low numbers of patients.

## Conclusion

The motivation of this study was to obtain an understanding of the current status of SRS/SRT in ANZ in a one day workshop attended by radiation oncology medical physicists around ANZ, in conjunction with departmental and workshop attendee opinion-based surveys. It is clear from the results of the departmental survey that there has been a rapidly increasing use of SRS/SRT around ANZ, most frequently using standard linac configurations.

From the workshop discussion, there was consensus that for a department to offer an SRS/SRT service, a minimum case load should be considered depending on availability of training, peer-review, resources and equipment. For example, this service may be limited to brain metastases only, with less common indications requiring more specialised equipment reserved for departments with a more comprehensive SRS/SRT program. Maintaining this equilibrium of referrals will lead to a balance between optimal SRS/SRT treatments and patient convenience, particular for those in rural/regional areas.

## Data Availability

The data that support the findings of this study are available from the corresponding author upon reasonable request.
